# Supplementary material for: The Effect of Continuous Intake of Lactobacillus gasseri OLL2716 on Mild to Moderate Delayed Gastric Emptying: A Randomized Controlled Study
Source: Nutrients. 2021 May 28;13(6):1852. doi: 10.3390/nu13061852 (PMC8230235; doi:10.3390/nu13061852)
Supplement: Supplementary file 1 [file nutrients-13-01852-s001.zip › Figure S1.pdf]

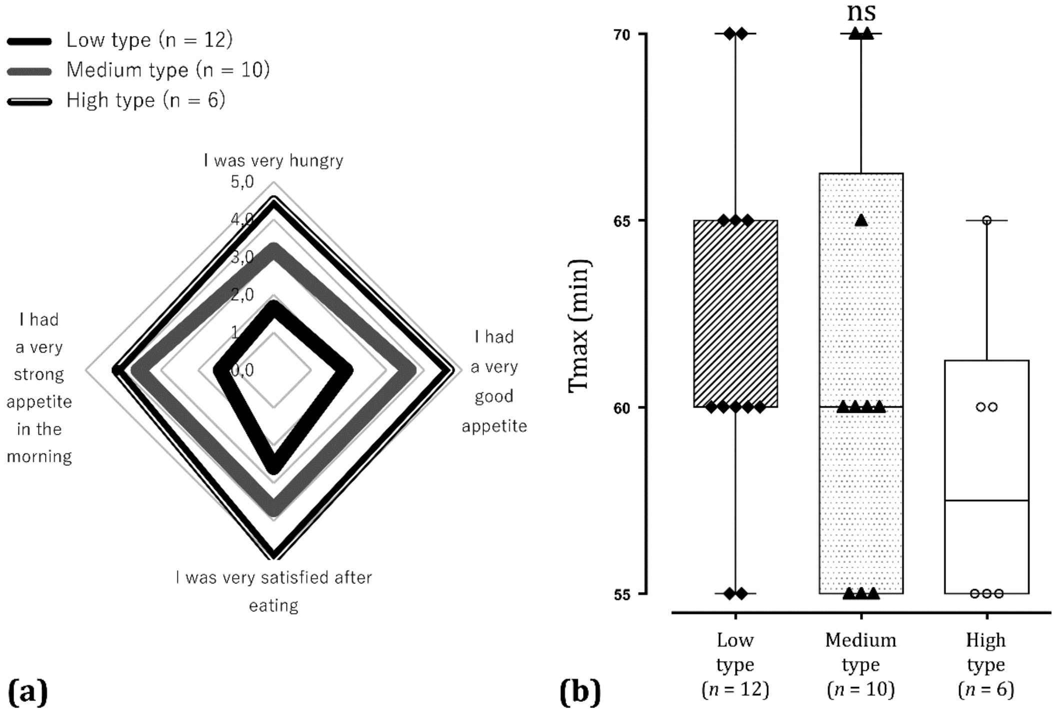

Figure S1. Participants' appetite types (before intake):

The participants' appetite types were classified using pre-intake data from the ITT population ($n = 28$). The appetite scores and Tmax for each type are shown in the figure. Appetite types were classified as follows based on the responses to the four questions on appetite shown in (a). Low appetite type: Those who answered between 0 (very untrue of me) and 2 (somewhat untrue of me) for two or more questions. High appetite type: Those who answered between 5 (true of me) and 6 (very true of me) for two or more questions. Medium appetite type: Those who did not fall into either the low or high appetite type.

(a) Radar chart of mean scores by appetite type (0, very untrue of me to 6, very true of me).

(b) Dot plots and box plots of Tmax by appetite type (median, 25th to 75th percentile; bars show minimum and maximum).

Intergroup comparison was performed using the Kruskal-Wallis test (significance level, $p < 0.05$ [two-sided]). ns, not significant; ITT, intention-to-treat.
