# Supplementary material for: The Effect of Continuous Intake of Lactobacillus gasseri OLL2716 on Mild to Moderate Delayed Gastric Emptying: A Randomized Controlled Study
Source: Nutrients. 2021 May 28;13(6):1852. doi: 10.3390/nu13061852 (PMC8230235; doi:10.3390/nu13061852)
Supplement: Supplementary file 1 [file nutrients-13-01852-s001.zip › Figure S2.pdf]

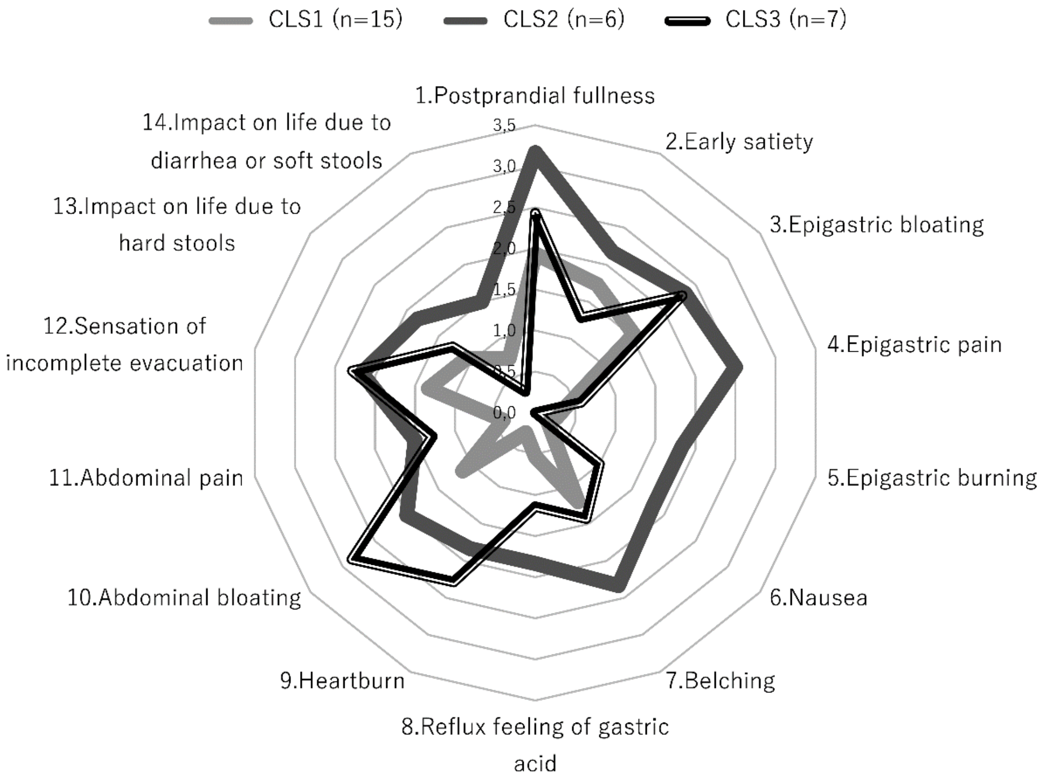

Figure S2. Participants' gastrointestinal symptom types (before intake):

Using the pre-intake data from the ITT population (n = 28), three factors were extracted from the severity of 14 general gastrointestinal symptoms using factor analysis. Participants were then classified into three clusters by hierarchical cluster analysis (Ward's method) based on these three factors. The mean scores for the 14 symptoms by cluster are shown in a radar chart.

Gastrointestinal symptom scores, 0 (none) to 6 (extremely severe).

ITT, intention to treat; CLS, cluster.
