# Supplementary material for: The Effect of Continuous Intake of Lactobacillus gasseri OLL2716 on Mild to Moderate Delayed Gastric Emptying: A Randomized Controlled Study
Source: Nutrients. 2021 May 28;13(6):1852. doi: 10.3390/nu13061852 (PMC8230235; doi:10.3390/nu13061852)
Supplement: Supplementary file 1 [file nutrients-13-01852-s001.zip › Figure S3.pdf]

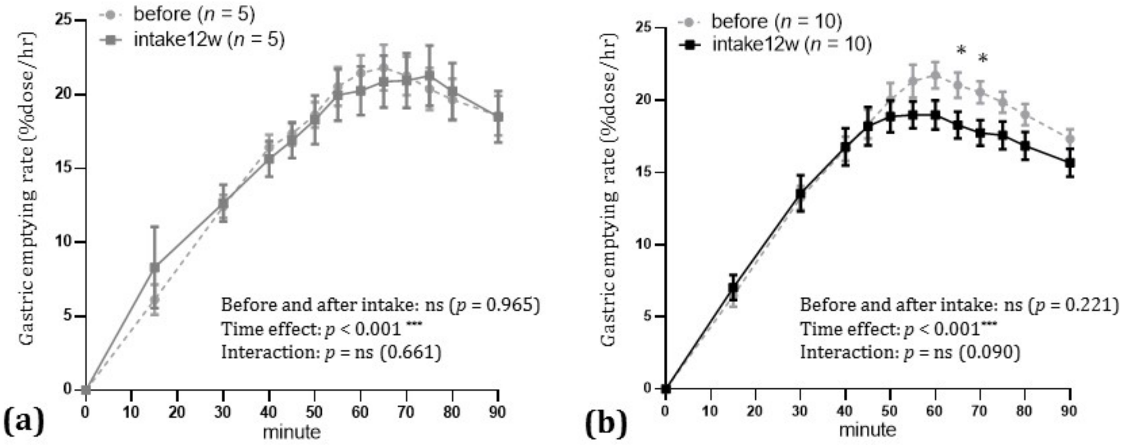

Figure S3. Effects of continuous intake of test food on gastric emptying rate following ingestion of a liquid meal in a strictly defined population of participants with mild to moderate delayed gastric emptying (excluding both high appetite and suspected pan-enteric dysmotility types). Gastric empty rates following ingestion of a liquid meal, as measured by the ^{13}C gastric emptying breath test performed before and 12 weeks after test food intake, are shown in the figure. The target populations were (a) placebo group and (b) LG21 strain group. Gastric emptying rate is shown as the mean ± standard error. Group effect (before and after intake), time effect, and interaction were tested using two-way repeated measures analysis of variance (significance level, $p < 0.05$ [two-sided]; *** $p < 0.001$ [two-sided]). Comparison of gastric emptying rate before and after intake at each time point was performed using Student t test (significance level, $p < 0.05$ [two-sided]; * $p < 0.05$ [two-sided]). ns, not significant.
